# Characterization of Zinc Oxide Nanoparticle Cross-Linked Collagen Hydrogels

**DOI:** 10.3390/gels6040037

**Published:** 2020-10-22

**Authors:** Yosra Agban, Odunayo O. Mugisho, Sachin S. Thakur, Ilva D. Rupenthal

**Affiliations:** 1 Buchanan Ocular Therapeutics Unit, Department of Ophthalmology, New Zealand National Eye Centre, Faculty of Medical and Health Sciences, The University of Auckland, Auckland 1142, New Zealand; y.agban@auckland.ac.nz (Y.A.); lola.mugisho@auckland.ac.nz (O.O.M.); 2 School of Pharmacy, Faculty of Medical and Health Sciences, The University of Auckland, Auckland 1142, New Zealand; s.thakur@auckland.ac.nz

**Keywords:** collagen, zinc oxide nanoparticles, cross-linking, mechanical properties

## Abstract

Collagen is the most abundant protein in mammals and possesses high biocompatibility and low antigenicity. These biological properties render it one of the most useful biomaterials for medical applications. This study investigated the mechanical and physical characteristics of collagen hydrogels cross-linked with different ratios of polyvinylpyrrolidone capped zinc oxide nanoparticles (ZPVP). Fourier transform infrared spectroscopy indicated molecular interactions between collagen fibers and ZPVP. Texture analysis revealed a significant increase in gel hardness, adhesiveness, and viscosity after cross-linking with ZPVP. Rheological measurements showed that as the ratio of ZPVP increased, stronger hydrogels were formed which in turn resulted in more sustained release of the model drug, dexamethasone sodium phosphate. We can therefore conclude that the mechanical properties of collagen hydrogels can be modified by controlling the ratio of ZPVP used for cross-linking, offering the potential to develop biocompatible sustained release drug delivery systems.

## 1. Introduction

Collagen is a natural protein polymer that constitutes up to 30% of all proteins in the human body [1]. It has an excellent biocompatibility and safety profile compared to synthetic polymers and has been used extensively in a wide range of biomedical applications [2]. Collagen is a major constituent of skin and musculoskeletal tissue and is a vital mechanical component for maintaining body strength and protecting the body from external stimuli. At a cellular level, it also provides a biological space for cell replication and differentiation that is essential for organogenesis and wound repair [3]. Such biological properties render collagen an ideal material for tissue regeneration and replacement, with successful use as a bone substitute, for skin replacement, and in the construction of artificial blood vessels and valves [4,5,6,7]. 

Biopolymers such as collagen can be easily formulated into solutions, dispersions, and nanoparticles, all of which have been used as drug delivery systems in ophthalmology [8]. Collagen is also a major constituent of ocular tissues and has been used in grafts for corneal replacement, bandage lenses, inserts, and shields for drug delivery to the corneal surface or intraocularly [9,10,11]. Drugs can be incorporated into collagen systems by simple entrapment as well as hydrogen or covalent bonding. Moreover, modifying the collagen structure by cross-linking can reduce its water uptake and slow down diffusion of any incorporated molecule [12]. Furthermore, interactions between the drug and collagen can also prolong drug release. 

The main disadvantages of simple collagen hydrogels include their poor mechanical properties and fast biodegradability, both of which can be overcome by combining collagen with other polymers or performing cross-linking [13]. Chemical cross-linking with aldehydes, isocyanates, and carbodiimides is commonly used; however, this has often been found to reduce the biocompatibility [13,14,15]. Metallic nanoparticles, on the other hand, can induce physical as well as photochemical cross-linking upon exposure to ultraviolet light [16,17]. The addition of polyvinylpyrrolidone capped zinc oxide nanoparticles (ZPVP) into the collagen matrix has previously been shown to induce gelation without the addition of chemical cross-lining agents [18,19]. The aim of this study was to compare the mechanical and physical properties of collagen hydrogels, rather than films for topical application as previously investigated [19], before and after cross-linking with different ZPVP concentrations and to determine if Col/ZPVP hydrogels can be used as injectable formulations for sustained drug delivery.

## 2. Results and Discussion

### 2.1. Visual Appearance

**Figure 1:**
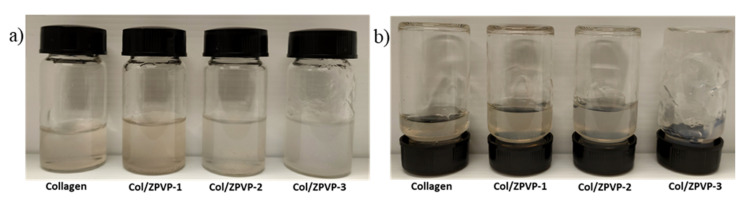
(**a**) Physical appearance of collagen, Col/ZPVP-1 (1:0.25), Col/ZPVP-2 (1:0.5), Col/ZPVP-3 (1:1) and (**b**) flow behavior when vials are inverted by 180°. Images were taken at room temperature.

Figure 1a shows the visual appearance of an unmodified collagen solution (left) and collagen solutions mixed with various amounts of ZPVP so that the final weight ratio of collagen to ZPVP was 1:0.25 (Col/ZPVP-1), 1:0.5 (Col/ZPVP-2) and 1:1 (Col/ZPVP-3). Figure 1b reveals that when inverting the vials by 180°, the unmodified collagen hydrogel (left) can be categorized as a solution. While the addition of ZPVP at ratios of 1:0.25 and 1:0.5 increased the viscosity, the hydrogels still remained in the solution state. However, when the concentration of ZPVP was increased to a ratio of 1:1 (Col/ZPVP-3), a partially dispersed viscous gel was formed.

### 2.2. Texture Analysis

**Figure 2:**
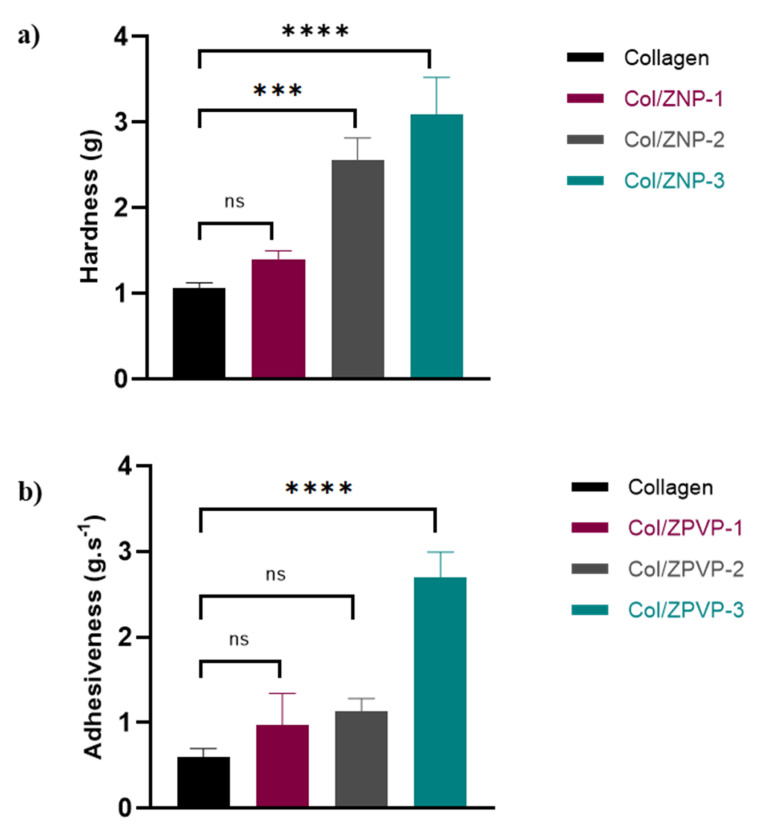
(**a**) Hardness and (**b**) adhesiveness of plain collagen, Col/ZPVP-1, Col/ZPVP-2, and Col/ZPVP-3 hydrogels at room temperature. Error bars represent the standard deviation (SD); *n* = 3; ns = non-significant (*p* > 0.05); *** *p* ≤ 0.001; **** *p* ≤ 0.0001.

Texture analysis is a useful tool to characterize hydrogels, with properties such as hardness and adhesiveness indicating ease of application and formulation retention at the site of administration [20]. Both hardness and adhesiveness increased in a linear fashion with increasing ZPVP concentrations (Figure 2a,b). Unmodified collagen had a hardness of 1.07 ± 0.06 g, which increased significantly (*p* < 0.001) after the addition of ZPVP to 2.57 ± 0.25 g and 3.10 ± 0.42 g for hydrogels Col/ZPVP-2 and Col/ZPVP-3, respectively. No significant difference in hardness was apparent between plain collagen and hydrogel Col/ZNPVP-1, which showed a hardness of 1.40 ± 0.10 g. 

An increase in adhesiveness was also seen for all hydrogels after the addition of ZPVP. Initially, collagen showed an adhesiveness of 0.60 ± 0.10 g.s^−1^ which increased to 0.97 ± 0.38 g.s^−1^ in hydrogel Col/ZPVP-1, 1.13 ± 0.15 g.s^−1^ in Col/ZPVP-2, and 2.7 ± 0.3 g.s^−1^ in Col/ZPVP-3. However, only the highest ZPVP ratio in Col/ZPVP-3 resulted in a significant (*p* < 0.0001) increase in adhesiveness when compared to the plain collagen hydrogel.

### 2.3. Syringeability

**Figure 3:**
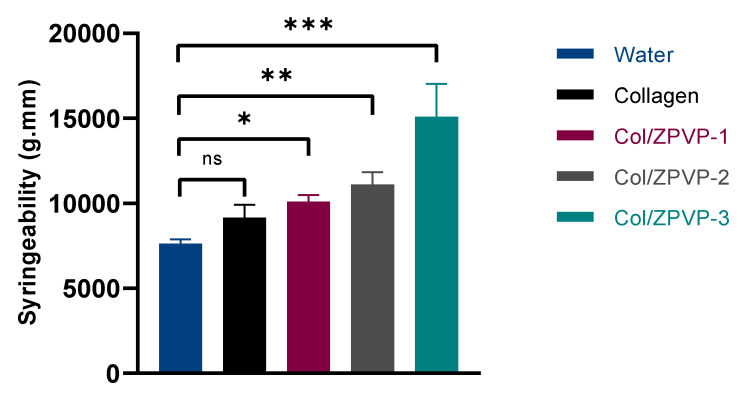
Syringeability of pre-filled syringes of collagen, Col/ZPVP-1, Col/ZPVP-2, Col/ZPVP-3, and water at room temperature. Error bars represent SD; *n* = 3; ns = non-significant (*p* > 0.05); * *p* ≤ 0.05; **p ≤ 0.01; *** *p* ≤ 0.001.

Syringeability is the force required to expel solutions or suspensions out of a syringe via a needle of predetermined gauge and length at a given injection rate. When hydrogels are used as injectable formulations, syringeability helps determine the ease and accuracy of administration. Figure 3 compares the syringeability of the various hydrogels to that of water, being the ideal reference for injectable formulations. The displacement rate of the gels was 1 mm/s which is equivalent to 4 mL/min, being in the acceptable injection flow rate range of 0.4 to 4 mL/min [21]. As expected, all of the pre-filled collagen syringes required a much higher force to push the plunger when compared to water. No significant (*p* > 0.05) difference was seen between unmodified collagen (9155 ± 445 g/mm) and water (7633 ± 149 g/mm); however, there was a direct proportional increase in application force required after the addition of ZPVP. The force required to expel the collagen-ZPVP hydrogels increased significantly to 10,111 ± 219 g/mm (Col/ZPVP-1; *p* < 0.05) and 11,106 ± 426 g/mm (Col/ZPVP-2, *p* < 0.01), respectively, while almost doubling to 15,089 ± 1,115 g/mm for Col/ZPVP-3 (*p* < 0.001). This may be a disadvantage as the high force required may cause irritation at the injection site and could more easily lead to dosing errors. As such, the higher ZPVP ratio hydrogel may be more suitable for formulating drug eluting films or solid implants, rather than being used as an injectable formulation. 

### 2.4. Rheological Evaluation

**Figure 4:**
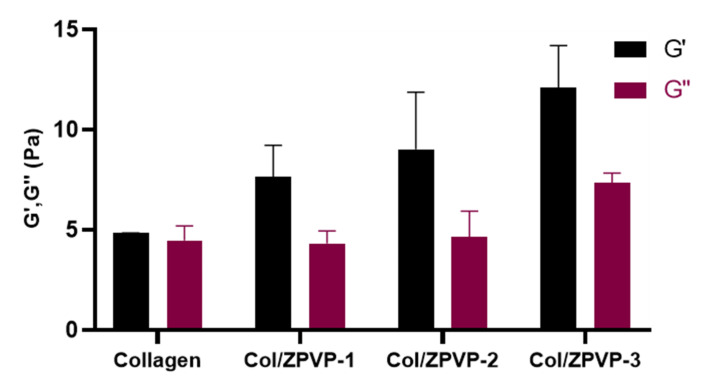
Storage (G′) and loss moduli (G″) from parallel plate rheometry at an angular frequency of 25 rad/s under 1% strain at room temperature. Error bars represent SD, *n* = 3.

The viscoelasticity of the collagen hydrogels was evaluated by measuring the storage (G′) and loss (G″) moduli, as shown in Figure 4. After the addition of ZPVP, G′ was found to be much higher than G″, with a directly proportional increase of G′ with increasing ZPVP ratios while G″ remained relatively constant. This is in agreement with the texture analysis results and confirms the strong viscoelastic behavior of the collagen hydrogels. It should be noted that we only examined a very narrow ZPVP concentration range and that higher ZPVP concentrations may in fact also result in much stronger hydrogels with an increased G″ [18].

### 2.5. FTIR Spectroscopy

**Figure 5:**
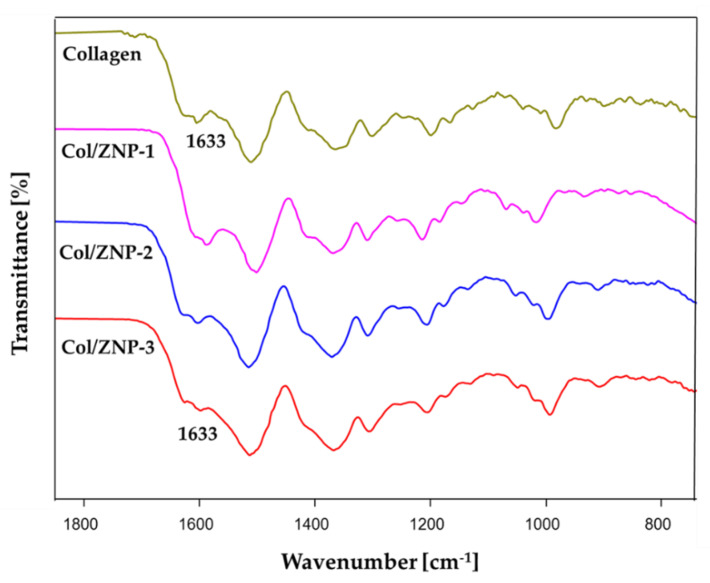
FTIR spectra of collagen, Col/ZPVP-1, Col/ZPVP-2, and Col/ZPVP-3.

The FTIR spectrum of collagen has five characteristic bands as shown in Table 1 [22]. Figure 5 shows that the position of all characteristic bands remained unchanged in both collagen and cross-linked collagen hydrogels. This indicates that ZPVP does not affect the molecular structure of collagen, which is favorable as it can still maintain its intrinsic properties [16]. However, as the concentration of ZPVP in the hydrogels increased, a slight decrease in the intensity of the amide I band was seen, suggesting H-bond interactions with the amide C=O group of the adjacent collagen molecule [18]. Lower amide I absorption is a result of the lower electron density when stronger hydrogen bonds involving the amide C=O are formed [16,23].

### 2.6. In-Vitro Drug Release

Dexamethasone is a potent glucocorticoid commonly prescribed in the treatment of a variety of inflammatory conditions. In ophthalmology, it is used in the management of both anterior and posterior segment inflammation including allergic conjunctivitis, keratitis, choroditis, and panuveitis [24,25]. Postoperatively, it is also prescribed as an immunosuppressive agent. However, dexamethasone has a relatively short half-life and therefore requires frequent administration, whether via topical drops for anterior eye conditions or intravitreal injections for posterior segment inflammation [26]. This can reduce patient compliance and may result in suboptimal treatment outcomes. Sustained delivery systems prolong the duration of drug release and decrease the dosing frequency which often results in improved therapeutic outcomes [27,28].

**Figure 6:**
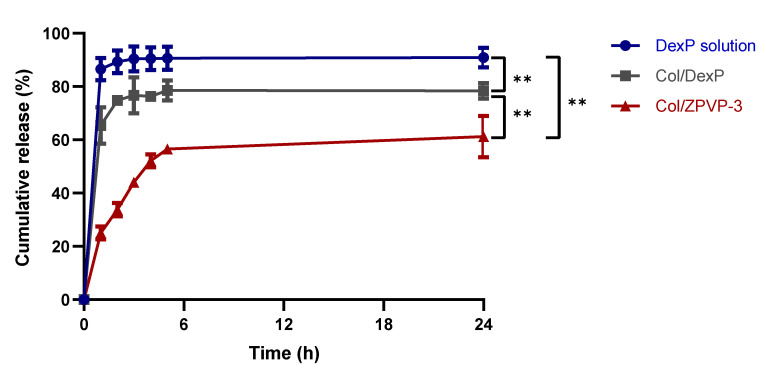
In vitro DexP release profiles in PBS (pH 7.4) at 37 °C from a DexP solution, Col/DexP and Col/ZPVP-3. Error bars represent SD, *n* = 3; ns = non-significant (*p* > 0.05); ** *p* ≤ 0.01.

In this study, dexamethasone sodium phosphate (DexP), a soluble salt form of dexamethasone, was used as a model drug to determine how the mechanical properties of the collagen hydrogels affected drug release. Figure 6 compares the cumulative DexP release from Col/DexP and Col/ZPVP-3 hydrogels to that from a DexP solution. While all formulations showed a similar biphasic profile with an initial burst release, followed by a slower drug release phase, the overall DexP release was significantly (*p* ≤ 0.01) slowed down after incorporation into unmodified collagen or the ZPVP cross-linked collagen to DexP solution. Within the first hour, the initial DexP burst significantly reduced from 86.5 ± 4.1%, as seen with the solution to 65.4 ± 6.9% (*p* = 0.0042) from Col/DexP and 26.0 ± 2.5% (*p* < 0.0001) from the Col/ZPVP-3 hydrogels. After 24 h, Col/ZPVP-3 still retained over 40% of DexP whereas Col/DexP had less than 20% and DexP of the drug remaining in the hydrogel. Overall, cross-linking collagen to ZPVP led to a significantly (*p* ≤ 0.01) more sustained drug release over 24 h when compared to DexP in solution or incorporated into the unmodified collagen hydrogel.

## 3. Conclusions

The use of collagen as a biomaterial in medical applications and drug delivery systems remains comprehensive due to its biological and mechanical properties. This study revealed that hardness, adhesiveness, syringeability, and rheological properties of collagen hydrogels are all directly correlated to the added ZPVP concentration, with the metallic nanoparticles resulting in physical cross-linking of the collagen fibers. Overall, modification of the mechanical hydrogel properties offers the opportunity to tailor and sustain drug release, which may ultimately result in better therapeutic outcomes when using such gels for biomedical applications.

## 4. Materials and Methods

### 4.1. Chemicals

Collagen and ZPVP (6 ± 2 nm), prepared as previously described [18], were supplied by the Leather and Shoe Research Association of New Zealand (Palmerston North, New Zealand).

### 4.2. Visual Apperance

The physical appearance of all collagen hydrogels was evaluated at room temperature. The mixtures were placed in glass vials and then categorized as gels or viscous solutions after inverting the vials by 180°.

### 4.3. Preparation of Collagen Hydrogels

Three mL of a 1.6% *w*/*w* collagen solution was placed in a glass vial. Adequate amounts of a 16 mg/mL stock suspension of ZPVP were added to the collagen solution so that the collagen to ZPVP weight ratios were 1:0.25 (Col/ZPVP-1), 1:0.5 (Col/ZPVP-2), and 1:1 (Col/ZPVP-3). The final concentration of collagen was 0.8% in all gel mixtures. The hydrogels were then shaken to ensure uniform distribution of ZPVP and then incubated overnight at 2–8 °C for gelation to occur.

### 4.4. Texture Analysis

Texture profile analysis was performed using a TA-XT Plus texture analyzer (Stable Micro Systems Ltd., Godalming, UK). A 10 mm diameter probe used in compression mode penetrated each sample at a speed of 1 mm/s to a depth of 5 mm and was subsequently withdrawn at a speed of 1 mm/s. The maximum force required to induce gel deformation during probe compression (hardness) and the maximum force involved during probe withdrawal from the gel (adhesiveness) were recorded and used to determine the mechanical properties of the hydrogels. All tests were carried out at room temperature in triplicate.

### 4.5. Syringeability

The syringeability of the hydrogels was determined using the TA-XT Plus texture analyzer (Stable Micro Systems Ltd., Godalming, UK). After preparation, gels were stored in 1 mL syringes (Becton, Dickinson and Company, Franklin Lakes, NJ, USA) equipped with a 27 G needle (length 13 mm, diameter 0.4 mm) at 2–8 °C for 24 h. Gels were then left to equilibrate at room temperature for 2 h before testing. All tests were performed in triplicate using the compression mode where the syringe plunger was pushed down a distance of 10 mm at 1 mm/s.

### 4.6. Rheological Evaluations

The dynamic viscoelastic functions (G´ and G″) were measured using a Discovery HR-2 rheometer (TA Instruments, New Castle, DE, USA). Measurements were performed using a 20 mm cross-hatched parallel plate geometry in order to prevent hydrogel slip. After loading the sample onto the rheometer plate, the gap was adjusted to 1 mm. Frequency sweeps were performed at an angular frequency ranging from 0.1 to 25 rad/s under 1% strain at room temperature to determine the linear rheological properties of the gels.

### 4.7. FTIR Spectroscopy

The infrared spectra of the hydrogels were recorded on a Bruker Tensor 37 spectrometer (Bruker Optics Inc, Billerica, MA, USA) using a diamond attenuated total reflection system. All spectra were recorded from 400 to 4000 cm^−1^ with over 128 scans at 2 cm^−1^ resolution.

### 4.8. In Vitro Drug Release 

Three formulations with an equivalent amount of 0.5% *w*/*v* of DexP were prepared: DexP solution, Col/DexP, and Col/ZPVP-3. Subsequently, 1 mL of each formulation was enclosed in a pre-swollen dialysis bag (MWCO 12,000-14,000 Da, Medicell International Ltd., London, UK) and suspended in 15 mL tubes filled with 8 mL of PBS (pH 7.4). The tubes were placed in a thermomixer (Eppendorf, Hamburg, Germany) maintained at 37 °C and 300 rpm. At predetermined timepoints, 0.5 mL of release medium was withdrawn and replaced with an equal volume of fresh PBS to maintain sink conditions and the drug content was analysed via HPLC [29].

### 4.9. Statistical Analysis

Statistical analysis was carried out using one-way analysis of variance (ANOVA) with Dunnett’s multiple comparison test and a paired *t*-test using GraphPad Prism 8 software. All p-values were considered significant if *p* ≤ 0.05 (ns: *p* > 0.05; * *p* ≤ 0.05; ** *p* ≤ 0.01; *** *p* ≤ 0.001; **** *p* ≤ 0.0001).

## Figures and Tables

**Table 1:** Characteristic collagen bands in FTIR spectra.

Band	Wavelength (cm^−1^)
Amide A	3305
Amide B	3018
Amide I	1633
Amide II	1552
Amide III	1239
